# Palliative Care Needs and Clinical Features Related to Short-Term Mortality in Patients Enrolled in a Heart Failure Unit

**DOI:** 10.3390/healthcare10091609

**Published:** 2022-08-24

**Authors:** Marta Aguilar-Fuerte, Fernando Alonso-Ecenarro, Alejandro Broch-Petit, Elena Chover-Sierra

**Affiliations:** 1 Nursing Department, Facultat d’Infermeria i Podologia, Universitat de València, 46010 València, Spain; 2 Internal Medicine, Consorcio Hospital General Universitario de Valencia, 46014 València, Spain; 3 Nursing Care and Education Research Group (GRIECE), GIUV2019-456, Nursing Department, Universitat de Valencia, 46010 València, Spain

**Keywords:** heart failure, palliative care, prognosis, comprehensive management, assessment, elderly

## Abstract

(1) Background: Heart failure (HF) is a chronic and complex pathology requiring continuous patient management due to clinical instability, associated comorbidity, and extensive pharmacological treatment. Its unpredictable course makes the advanced stages challenging to recognize and raises the need for palliative care. This study aims to identify palliative care needs in HF patients and describe clinical features related to short-term mortality. (2) Methods: A descriptive, observational, cross-sectional, and retrospective study was carried out in an HF unit of a Spanish tertiary hospital. Patients’ socio-demographic and clinical data were collected from clinical records, and different instruments were used to establish mortality risks and patients’ needs for palliative care. Subsequently, univariate and bivariate descriptive analyses were performed. A binary logistic regression model helped to determine variables that could influence mortality 12 months after admission to the Unit. (3) Results: The studied population, sixty-five percent women, had an average age of 83.27 years. Among other clinical characteristics predominated preserved ejection fraction (pEF) and dyspnea NYHA (New York Heart Association) class II. The most prevalent comorbidities were hypertension and coronary heart disease. Forty-nine percent had a low–intermediate mortality risk in the following year, according to the PROFUND index. The NECPAL CCOMS-ICO© instrument identified subjects who meet the criteria for palliative care. This predictive model identified NECPAL CCOMS-ICO© results, using beta-blockers (BB) or AIIRA (Angiotensin II receptor antagonists) and low glomerular filtration rate (GFR) as explanatory variables of patients’ mortality in the following year. (4) Conclusions: The analysis of the characteristics of the population with HF allows us to identify patients in need of palliative care. The NECPAL CCOMS-ICO© instrument and the PROFUND have helped identify the characteristics of people with HF who would benefit from palliative management.

## 1. Introduction

Heart failure (HF) is a chronic, progressive syndrome and a final common pathway of various diseases in which a decrease in heart function occurs [1]. About 1–2% of the adult population in developed countries suffers HF, with this percentage rising as age increases, reaching more than 10% of the population over 70 years and up to almost 20% if they are above 80 years old [2].

The evolution of this syndrome leads to the onset of signs and symptoms that decrease the patient’s functional capacity, loss of independence, an increase in the number of visits to emergency rooms, hospital admissions, and finally, death from refractory HF. Even so, and despite many advances in the treatment of HF, about 40% of patients die in the year after their first hospital admission [3,4].

HF is a chronic and complex pathology that requires continuous management due to the clinical variability in the patient. Its evolutionary course is challenging to predict. Consequently, patients in an advanced stage are frequently not identified or, if so, are in advanced stages of the disease when it is too late. On many occasions, neither the patient nor the family feels that it is a terminal illness, nor do the health professionals, who do not recognize the proximity of death, which means that the patient ends up dying with active treatment which is not always necessary [5,6].

Palliative care is poorly developed in heart disease. The main reason could be the considerable uncertainty regarding its prognosis because of the unpredictable trajectory that characterizes this disease, which leads to exacerbations and the subsequent improvement of the symptoms multiple times before death [7,8]. Different organizations have developed guidelines for managing advanced HF and identifying patients with HF who would benefit from this palliative management. Even so, there are significant differences between territories in providing palliative care to people with HF, and few specific resources have been developed for this population [3,9,10,11].

Both physical and emotional symptoms cause significant alteration in patients’ and caregivers’ quality of life, which is usually not recognized and even less treated [5]. Patients and caregivers usually face a decision-making process regarding the introduction of advanced therapies and even regarding advanced life support measures, with limited knowledge about their effects in the disease course, mainly during episodes of decompensation and hospitalization [11,12,13].

The following paper presents a study carried out in a Heart Failure Unit, included in the UMIPIC program of the Spanish Society of Internal Medicine, based on the comprehensive care of elderly patients with HF and other comorbidities [14,15,16]. This program, made for managing patients with HF and other comorbidities, is extended throughout Spain. There are more than 30 units with similar functioning, following the protocols established by the Spanish Society of Internal Medicine working group.

Nurses and internists who specialize in managing patients with HF work in these units. A model for the management of chronic patients with HF is proposed, centered on three main aspects: the education of basic concepts involving the patient and their relatives in their disease, the comprehensive assessment of this type of patient, and the continuity of care in coordination with primary healthcare level. Its general objectives are to reduce the rate of readmissions and visits to the Emergency Department, reduce the number of visits to other specialties, reduce morbidity and mortality, and improve patients’ quality of life [15,16,17].

The study aimed to identify the socio-demographic and clinical characteristics of patients with chronic HF at the time of their enrollment in the UMIPIC follow-up program of a Spanish hospital and, therefore, to detect the need for palliative care using prognostic and survival criteria.

## 2. Materials and Methods

### 2.1. Design

A descriptive, cross-sectional, and retrospective study was carried out in the UMIPIC of Consorcio Hospital General Universitario in Valencia, Spain. 

### 2.2. Studied Population 

All patients who had been attended at least once in the Heart Failure Unit since its creation from 2012 to December 2018 were included in the study. 

The criteria to include a patient in the UMIPIC program (and so on in this study) are aged over 70, not having cognitive impairment, adequate family support with a diagnosis of HF, and other comorbidities. Besides, they should have had at least one episode of hospitalization or been attended to in the emergency room because of HF in the previous six months (acute decompensation solved in the emergency room (mild) or needing hospitalization).

### 2.3. Data Collection

All the information necessary to carry out this work was collected by reviewing the medical records of the patients who had ever come to the Unit. All the necessary data are part of the information collected in their first visit to the Unit (during nurse’s and physician’s interview or physical exploration and from the results of complementary exams (blood tests)) or could be calculated (as in the case of some indicators) from said information. For this reason, and based on the records of the Unit on the patients included in it since it was put into operation, this information was collected from the computerized clinical records of each patient where it was available. All the authors, members of the Internal Medicine service or of the UMIPIC itself, could access the existing records in the computerized clinical history.

The clinical and socio-demographic data collected were: (i) age, (ii) gender, (iii) cohabitants of the patient (living alone, with their couple, with a non-familiar caregiver, with another familiar caregiver, or living in a nursing home), (iv) their way of accessing the Unit (after a hospitalization episode, derivated from their Primary Healthcare Center, from the emergency unit or other medical specialist al cardiologist or nephrologist), (v) their LVEF (preserved, mildly-reduced or reduced HF), (vi) presence of edema in lower extremities, (vii) drugs used in HF management, (viii) comorbidities, (ix) number of attendances in the emergency room or (x) hospitalizations related to HF in the last year, and (xi) biomarkers’ serum levels (proBNP, sodium and potassium, urea, creatinine and Glomerular Filtration Rate (GFR), ferritin and transferrin saturation index among others). Moreover, it was considered whether the patient’s death had occurred before the first year of their inclusion in the UMIPIC program. This information on the death (or not) was collected from the information offered by the population information system of the Valencian Community, where the date of death appears, provided that it took place in the Community and independently of the place of death. For this reason, patients included in this Unit were selected until December 2019 since data collection began in January 2020, and we wanted to collect data from patients who had been in follow-up for at least one year (or who had died in that first year).

In addition to clinical and socio-demographic data, information was collected on cognitive impairment (Pfeiffer test) and patient’s level of dependency (Barthel index), both evaluated on that first visit.

Finally, using the existing data in the clinical record, three indicators were used to evaluate the probability of mortality and palliative care needs. These indicators are calculated from clinical and socio-demographic data and are the following:

- Charlson comorbidity index (CCI). System of evaluation of life expectancy in 10 years, depending on the age and comorbidities of the subject. It collects information on 19 pathologies whose presence has been shown to influence the reduction in life expectancy. It provides a numerical score and an estimated 19-year survival percentage [18,19].
- PROFUND Index. It is an instrument to predict annual mortality risk in pluripathological patients [20]. It assesses clinical and socio-demographic data. Its score ranges from 0 to 30, and a higher score indicates a higher probability of death the following year; four levels of death risk are established according to this score [21].
- NECPAL CCOMS-ICO©. An instrument for the early identification of people with palliative care needs. It draws from the so-called “surprise question” (SQ) addressed to the professional, “Would you be surprised if this patient died in the next 12 months?”. If the healthcare professional answers “no” to this question, the patient is considered SQ+. A SQ+ patient is also NECPAL+ when presenting at least one additional parameter from the NECPAL CCOMS-ICO© tool (request or need for PC; general clinical indicators of severity and progression, including comorbidity and resource use; and disease-specific indicators). A patient classified as NECPAL+ requires palliative care [22,23,24].

### 2.4. Data Analysis

Univariate descriptive analysis was performed, presenting the quantitative variables using measures of central tendency and the categorical ones using frequency distribution. Subsequent bivariate analysis was used to assess the correlation among numerical variables using Spearman’s non-parametric test and to analyze mean differences with Kruskal–Wallis or Mann–Whitney non-parametric tests. Moreover, the chi-square test was used to study relationships between two categorical variables.

Lastly, a binary logistic regression model was developed to analyze the probability of death in the first 12 months after the inclusion of the participants in the program. This model comprises all those variables, both quantitative and qualitative, which showed statistically significant relationships with that death before that period.

To select the most suitable model, the hierarchical model comparison procedure was used, which is based on the suppression in a sequential way (one at a time) of those variables and interactions whose effects had higher and not significant values in the Wald test.

The software © IBM © SPSS Version 24 for © Microsoft Windows was used for statistical analysis.

Statistical significance was established at *p* < 0.05 in all cases, with a 95% confidence interval.

### 2.5. Ethical Aspects

Every patient who enters the Unit and the UMIPIC program signs an informed consent, establishing the possibility of using clinical record data for research purposes, whereby at the time of admission to the program, the patient or legal/procedural representative is aware and accepts the condition. This informed consent, elaborated by the bioethical section of the Spanish Society of Internal Medicine, is included in the report on the creation of each UMIPIC unit, and the CHGUV ethics committee approved the study in March 2018.

Besides, all patients’ data were extracted from the consolidated clinical record since access to professionals at the center is allowed and were conveniently anonymized before their analysis.

## 3. Results

### 3.1. Characteristics of the Studied Population

Finally, data were collected from 408 patients, primarily women, with an average age of 83.27 years, with an age range between 70 and 97. Most of them lived with a caregiver from a non-family setting. The rest of the socio-demographic and clinical characteristics are shown in Table 1.

### 3.2. Results of Prognostic Instruments/Tools for Mortality Risk and the Need for Palliative Care

Table 2 shows the results of the instruments used to assess both the probability of proximal death (CCI and PROFUND) and the need for palliative care (NECPAL CCOMS-ICO©).

Notably, the Charlson index shows the high comorbidity of the studied population and, therefore, their low probability of surviving more than ten years on. Moreover, according to the PROFUND index, 8% of people were likely to die in the following year. Almost a third of the population could be considered in need of palliative care (all patients with SQ+ have at least one indicator, so they were NECPAL+).

The relationships between the three instruments were studied, finding only a statistically significant linear relationship between the number of indicators of the NECPAL CCOMS-ICO© instrument and the Charlson index (rho = 0.64, *p* = 0.000) but not between the NECPAL CCOMS-ICO© indicators and the PROFUND index (rho = 0.16, *p* = 0.13) or between PROFUND and Charlson (rho = 0.13, *p* = 0.38).

It was also seen that patients with NECPAL+, whose death during the following year would not surprise professionals, had higher results both in Charlson (8.86 ± 1.62 vs. 6.70 ± 1.33) and in PROFUND (7.79 ± 4.42 vs. 4.47 ± 2.58), statistically significant differences (*p* = 0.000 in both cases).

### 3.3. Relationship among the Socio-Demographic and Pathological Characteristics of Population and Measurement Instruments with Mortality before Twelve Months of Admission to the Unit 

When analyzing clinical records, it was found that 13% of the patients died in the first year after their admission to the unit, so we tried to identify differential characteristics between those who died in that first year and those who did not.

Regarding pharmacological treatment, it has been observed that 81.6% of those who did not die in the first year took beta-blockers (BB), while 53.85% of those who died did not take them. This relationship was statistically significant according to the chi-square test (*p* = 0.005). In the case of angiotensin II receptor antagonists (AIIRA), it was found that they were used by 44.8% of the subjects who did not die, while only 15.4% of the subjects who died before 12 months took them. This relationship was also statistically significant (*p* = 0.044 in the chi-square test).

Regarding comorbidities, it is noted that 61.5% of the subjects who died during the first year and only 33% of those who did not were diagnosed with COPD (*p* = 0.049 in the chi-square test). As for CKD, 63.2% of the participants who did not die in the first year after entering the Unit did not suffer it, but 77% of those who died have this comorbidity (*p* = 0.006 in the chi-square test). Furthermore, the GFR rate was significantly lower in the subjects who died in that first year (29.03 ± 13.86 vs. 49.66 ± 20.71) (*p* = 0.001 Mann–Whitney test).

Regarding the rest of the biomarkers’ values, only statistically significant differences were found in the proBNP values, higher in the group of people who died before the first year (5933.33 ± 3606.53 vs. 2880.46 ± 3953.29) (*p* = 0.001 Mann–Whitney test). Although the differences were not statistically significant, people who died before the first year also had lower hemoglobin values (11.88 ± 1.8 vs. 11.91 ± 1.99) (*p* = 0.84 Mann–Whitney test), ferritin (136.85 ± 117.91 vs. 178.06 ± 247.03) (*p* = 0.87 Mann–Whitney test) and Transferrin saturation (17.20 ± 7.99 vs. 25.82 ± 34.98) (*p* = 0.30 Mann–Whitney test).

Measurements made with the instruments used to predict mortality, prognosis, and survival have shown statistically significant results when relating them to death within 12 months of inclusion in the Unit. Thus, it is observed that people who died in the first year had higher comorbidity, measured with the Charlson index (7.08 ± 1.64 vs. 9 ± 1.35) (*p* = 0.001 Mann–Whitney test).

The results of the PROFUND index indicate that among the subjects who did not die before the first year, 25.3% were at low risk, and 47.1% were at a low-intermediate risk. In contrast, among the subjects who did die, 46.1% were of medium-high risk, and 23% were of high risk. According to the chi-square test (*p* = 0.041), this relationship was statistically significant. We also found statistically significant differences in the score obtained in PROFUND between both groups, which was higher in the group that died before the first year (8.54 ± 5.47 vs. 4.97 ± 2.93) (*p* = 0.03 Mann–Whitney test).

In the case of NECPAL CCOMS-ICO©, we found that 76.9% of the patients who died before the first year had a SQ+ (their death will not surprise the health professional), and in 78.2% of those who did not die, they had a SQ- (their death will surprise the health professional) (*p* = 0.000 in the chi-square test). Regarding the NECPAL CCOMS-ICO© tool indicators, we found a more significant number of prognostic indicators for PC in those who died before the first year (3.23 ± 0.93 vs. 2.24 ± 1) (*p* = 0.001 Mann–Whitney test).

### 3.4. Binary Logistic Regression Model, Explaining the Probability of Death before Twelve Months

Once those variables that were statistically related to the probability of dying in the first year were identified, these variables were used to develop the binary logistic regression model. Table 3 shows the coefficients corresponding to the statistically significant variables.

Thus, it is implied that the use of the drugs BB and AIIRA could be considered protective factors, as indicated by their negative coefficient sign. It can be said that, on equal terms, subjects who take BB have a 12.2% less probability of dying in the first year, and those who take AIIRA have 11.4% less.

The value of GFR would also be a factor related to the probability of death in the first year since those people with a lower GFR would have a greater probability of dying. For each unit that decreased the GFR, the subject had a 93.7% probability of death before a year.

Regarding the SQ from NECPAL CCOMS-ICO©, an indicator of the perception of the health professional who evaluates the patient, it is observed that those people whom the professional would be surprised if this happened are less likely to die within a year.

## 4. Discussion

Various studies describe the profile of patients with chronic HF focusing on socio-demographic, clinical, and analytical variables. However, fewer works are focused on the palliative care needs of those patients, such as the one presented here.

The profile of the patient treated in our Unit is that of a woman with an average age of 83 years, who associates considerable comorbidity, highlighting Type 2 Diabetes Mellitus and Hypertension and, to a lesser extent, ischemic heart disease. Most of them have HFpEF (>50%). This profile is similar to the one identified in other studies developed in Spain and different HF units [14,16,25,26,27,28].

The fact that there are more women than men in our population would also explain the lower presence of patients with a record of ischemic heart disease and, therefore, the lower percentage of patients with rEF heart failure. Studies discussing gender differences in HF show that in the population older than 70 years, the prevalence of HF is higher in women. Women’s mortality from chronic HF is lower than men’s, but their quality of life is worse [29]. Even against the therapy background, a significant limitation of functional capabilities remains. Hormonal, pathophysiological, and structural factors have been postulated as explanations for these gender differences, but much remains to be investigated [30,31]. We also know that HFpEF is higher in older age groups [32,33], which justifies the lower number of people with HFrEF among the studied population. Research is nowadays scarce, mainly due to the smaller number of women and older people included in clinical trials [34,35].

Furosemide and beta-blockers are among the most widely used drugs in our population. Considering that the majority of our patients are in NYHA Class II, it can be asserted that they follow an adequate treatment regimen as established in the current clinical practice guidelines [36]. The degree of dyspnea, when considering gender, showed that women with HF are more limited in this regard, presenting a higher percentage of individuals in NYHA classes III and IV, as current the literature has shown [32,37,38]. This could explain why women participating in this study present worse results in the Barthel index.

The instruments used to predict mortality have been the Charlson index and the PROFUND. This combination of instruments has been used in other studies in populations similar to those under study [20,39,40,41]. Furthermore, and since it is a retrospective study in which it is known which patients have died before the first year of admission to the Unit, a contribution of something more to the prognostic value of these instruments has been possible.

The studied population has shown a high comorbidity index (CCI ≥ 7.33), indicating in a significant way a probability of death in the first year of 85%, analogously to other studies with a similar profile of patients [22,25].

Regarding the PROFUND index, it is observed that 49% (21.5–31.5%) of patients show a low-intermediate risk of dying before the year, with 8% presenting a high risk. At 12 months after admission to the Unit, 13% died, showing significance in the estimation made by the PROFUND index (*p* = 0.041), making this a good indicator of mortality risk before the year, as other publications show. Investigations such as that of Díez-Manglano and de la Rica [21,42] demonstrate that the PROFUND index helps predict long-term global mortality in multiple pathology patients in Internal Medicine, showing increased mortality in patients with a score greater than 10.

When applying the NECPAL CCOMS-ICO© instrument, it is found that 29% of patients with a positive Surprise Question. These results are in line with the Gastelurrutia study [4], also conducted in Spain, in patients with HF. Furthermore, all the subjects presented at least one of the complexity parameters shown in the instrument, which allows identifying this percentage in our population that would meet the criteria for palliative care.

These results are similar to studies carried out with pluripathological patients, in which NECPAL CCOMS-ICO© has also shown this utility. Thus, there are different studies in which patient follow-up has been performed, comparing patients with positive and negative responses, showing significant statistical differences in mortality. The usefulness of the NECPAL CCOMS-ICO© instrument has been shown in different studies, in which the application of the NECPAL instrument shows which people are subsidiary to palliative care [43,44]. Furthermore, this work has verified the existence of a statistically significant relationship between the results in NECPAL CCOMS-ICO© and the fact that the patient died before 12 months of admission to our Unit, which results in the usefulness of NECPAL CCOMS-ICO© in identifying patients with HF who need palliative care.

It must be pointed out that the three instruments used in this study have been used in other studies, such as that of da Costa [45]. It has been shown that although the three instruments were related significantly to higher mortality, there was a low concordance among the three instruments. In this study, statistically significant relationships have been found among the three instruments. Even so, to the best of our knowledge, these three instruments have not been used together to study the characteristics of people diagnosed with HF. 

The binary logistic regression model developed to study the influence of the different explanatory variables on death in the first year after admission to the Unit shows that subjects treated with BB and ARAS and those with higher GF values are less likely to die. It also shows us the critical role played by the opinion of health professionals who evaluate patients, in this case, expressed through SQ, since those subjects with SQ+ are more likely to die.

The identifying role of the need for palliative care of the NECPAL CCOMS-ICO© instrument, the protective effect of drugs against mortality, and the importance of CKD as a comorbidity to control exhaustively because it increases mortality in people with HF are also confirmed in the studied population [46,47,48,49]. Thus, the identification and proper management of patients with CKD and the optimization of the pharmacological treatment of HF continue to appear as aspects to be highlighted in managing HF in our population to achieve better results, as recommended in the management guides for this pathology [1,5,50,51,52].

The main limitation of this work derives from the fact that the socio-demographic and clinical characteristics of patients from an HF Unit of a single Hospital (CHGUV) are presented. Hence, it is not going to be representative of the Spanish population suffering from HF. Nonetheless, it does allow us to get closer to their profile and, since they are old adults, delve into the aspects related to this identification of palliative care needs of our patients, which was set as the main objective of this study.

Another limitation is based on the type of design. By proposing a retrospective design and collecting the existing data on the clinical history, knowing what other variables outside the existing records may have influenced the mortality of the patients is not possible. From this point on, it is proposed to make a long-term follow-up of the new patients included in the Unit to establish a good follow-up of the subjects and deepen the need for palliative care in this chronic disease.

The possibility of identifying their needs for palliative care from the first contact of a patient with our HF unit allows us to better plan the phases of care and follow-up in our unit, which in many cases continues until the death of the patient or until the referral to specific palliative care resources.

The results of NECPAL and PROFUND can be added to the initial assessment of the patient in UMIPIC since most of the information collected in them is already included in the information collected in that first contact.

## 5. Conclusions

Identifying variables that significantly influence the short-term mortality of people with HF and other comorbidities could allow health professionals to optimize their management. The knowledge of those aspects with a more significant relationship with mortality in people with HF, such as the coexistence of renal disease and optimization of treatment to control blood pressure and heart rate, would improve the quality of life of these patients.

Identifying those patients who will benefit from a palliative care approach will allow different healthcare processes to be implemented. That would help reach people with advanced stage HF, a dignified end of life without pain, and receive proper attention to their death process, facilitating their quality of life.

The NECPAL and PROFUND instruments have shown their usefulness in identifying people with HF who are likely to benefit from a palliative approach to their management.

## Figures and Tables

**Table 1:** Clinical and socio-demographic characteristics of the studied population.

		*n*	%	Mean	SD
Age				83.27	5.67
Gender	Female	265	64.95		
	Male	143	34.05		
Cohabitants	Nobody	110	26.96		
	Couple	98	24.02		
	Non-familiar caregiver	122	29.90		
	Familiar caregiver	74	18.14		
	Nursing home	4	0.98		
LVEF	pEF	310	75.98		
	mEF	45	11.03		
	rEF	53	12.99		
NYHA	Class I	16	3.92		
(dyspnea classification)	Class II	285	69.85		
	Class III	98	24.02		
	Class IV	9	2.21		
Access to the HF unit	Post-hospitalization	314	76.96		
	Primary health care	33	8.09		
	Emergency department	29	7.11		
	Other specialists	32	7.84		
Edema in lower extremities	Yes	126	30.88		
	No	282	69.12		
Emergencies (in last year)	Two or less	196	48.04		
	More than two	212	51.96		
Hospitalizations (in last year)	Two or less	326	79.90		
	More than two	82	20.10		
Drugs used (number)				10.05	2.99
Drugs used (type)	Furosemide	404	99.02		
	Chlorthalidone	69	16.91		
	Spironolactone	78	19.12		
	Thiazides	37	9.07		
	Eplerenone	16	3.92		
	Beta-blockers	314	76.96		
	Digoxin	65	15.93		
	ACE Inhibitors	143	35.05		
	AIIRA	167	40.93		
	AVK	171	41.91		
	Direct oral anticoagulants	94	23.04		
Comorbidities	DM	196	48.04		
	HBP	388	95.10		
	Coronary heart disease	114	27.94		
	COPD	151	37.01		
	CKD	171	41.91		
	Stroke	69	16.91		
	Atrial Fibrillation	286	70.10		
	Valvulopathies	159	38.97		
Biomarkers’ values	Hemoglobin			11.91	1.98
	Hematocrit			39.71	33.80
	Ferritin			172.20	234.28
	Transferrin saturation			46.92	21.09
	GFR			24.70	32.85
	Pro-BNP			3262.07	40,007.26
Barthel index	Independent	135	33.03		
	Minimally dependent	200	49.04		
	Partially dependent	45	11.05		
	Very dependent	28	6.88		
Pfeiffer SPMSQ	Intact intellectual functioning	355	87.01		
	Mild intellectual impairment	49	12.01		
	Moderate intellectual impairment	4	0.98		

SD: Standard deviation; LVEF: left ventricle’s ejection fraction; pEF: preserved Ejection Fraction; mEF: mildly reduced Ejection Fraction; rEF: reduced Ejection Fraction; NYHA: New York Heart Association ACE: Angiotensin-converting enzyme; AIIRA: Angiotensin II receptor antagonist AVK: antivitamin K DM: Diabetes Mellitus; HBP: High blood pressure; COPD: Chronic obstructive pulmonary disease; CKD: Chronic Kidney Disease GFR: Glomerular Filtration Rate; BNP: Brain Natriuretic Peptide; SPMSQ: Short Portable Mental Status Questionnaire.

**Table 2:** Results of instruments used to assess mortality risk and palliative care needs.

		Mean	SD	*n*	%
CCI		7.33	1.75		
PROFUND Index (score)		5.44	3.53		
PROFUND Index	Low risk			90	22.06
(mortality risk levels)	Low-medium risk			200	49.02
	Medium-high risk			86	21.08
	High risk			32	7.84
NECPAL CCOMS-ICO© (SQ)	(SQ+)			118	28.92
	(SQ−)			290	71.08
NECPAL CCOMS-ICO© (Indicators)		2.37	1.04		

SD: Standard deviation; CCI: Charlson Comorbidity Index. S.Q.: Surprise Question.

**Table 3:** Variables and coefficients in the binary logistic regression model.

	Β	*p*-Value	OR	OR CI (95%)
Beta-blockers	−2.105	0.012	0.122	0.24–0.628
AIIRA	−2.175	0.034	0.114	0.015–0.849
GFR	−0.065	0.023	0.937	0.885–0.991
Surprise question	−2.362	0.007	0.094	0.017–0.531

OR: Odds Ratio; OR CI: Odds Ratio Confidence Interval.

## Data Availability

The data presented in this study are available on request from the corresponding author.
